# Enhancing the Accuracy of Measuring DEP Force Applied on Cells by Considering the Friction Effect

**DOI:** 10.3390/bios13050540

**Published:** 2023-05-12

**Authors:** Alireza Khouzestani, Yousef Hojjat, Marziyeh Tavalaee, Hesam Sadeghian, Mohammad Hossein Nasr-Esfahani

**Affiliations:** 1 Department of Mechanical Engineering, Tarbiat Modares University, Tehran 14115-175, Iran; a.khouzestani@modares.ac.ir (A.K.); hesam.sadeghian@modares.ac.ir (H.S.); 2 Department of Animal Biotechnology, Reproductive Biomedicine Research Center, Royan Institute for Biotechnology, ACECR, Isfahan 8165131378, Iran; m.tavalaee@royan-rc.ac.ir; 3 Isfahan Fertility and Infertility Center, Isfahan 8158858151, Iran

**Keywords:** Dielectrophoresis, microfluidics, sperm selection, force experimental measurement, friction

## Abstract

The Dielectrophoresis (DEP) phenomenon has been widely used for cell separation in recent years. The experimental measurement of the DEP force is one of the concerns of scientists. This research presents a novel method for more accurately measuring the DEP force. The innovation of this method is considered the friction effect, which has been neglected in previous studies. For this purpose, first, the direction of the microchannel was aligned with the electrodes. As there was no DEP force in this direction, the release force of the cells caused by the fluid flow equaled the friction force between the cells and the substrate. Then, the microchannel was aligned perpendicular to the direction of the electrodes, and the release force was measured. The net DEP force was obtained by the difference between the release forces of these two alignments. In the experimental tests, the DEP force, when applied to the sperm and white blood cell (WBC), was measured. The WBC was used to validate the presented method. The experimental results showed that the forces applied by DEP to WBC and human sperm were 42 pN and 3 pN, respectively. On the other hand, with the conventional method, these figures were as high as 72 pN and 4 pN due to neglecting the friction force. The compression between the simulation results in COMSOL Multiphysics and the experiments determined the new approach to be valid and capable of use in any cell, such as sperm.

## 1. Introduction

Among every six couples, at least one couple has experienced, at least once, some kind of infertility at a reproductive age [1]. In recent decades, different assisted reproductive techniques, such as intracytoplasmic sperm injection (ICSI) and in vitro fertilization (IVF), have been widely used to improve fertility rates. Identifying and sorting out the superior motile spermatozoa with DNA integrity is vital for successful IVF and ICSI. Considering the fragile nature of sperms, sorting out high-quality sperms using conventional techniques such as swim-up or the density gradient centrifuge (DGC) has been associated with several challenges, including the possibility of damaging the sperm’s DNA integrity and being time-consuming [2].

In recent years, microfluidic techniques have been continuously developed to separate superior motile spermatozoa with the best morphology and DNA integrity, and soon, they will replace conventional methods [3]. Microfluidic systems require less sample volume, which has several advantages, such as reducing the cost of materials, being environmentally friendly, and so forth [4]. In addition, the smaller size of the device and lower manufacturing costs are other advantages of such systems [5]. Chemotaxis [6], thermotaxis [7], electrophoretic [8,9], zeta-potential [10], and DEP [11] are among the active cellular selection methods that can separate healthy sperm in microfluidic sperm sorting actuators. Recently, DEP was implemented for the continuous transportation of a single cell [12]. However, it is mainly used in lab-on-a-chip devices for analyses and the separation of biological samples [13]. DEP actuators use the difference in electrical properties for separation. The advantage of these actuators is that they can separate particles with high purity and efficiency. Additionally, DEP is a label-free method [14,15]. DEP is a force applied on a polarizable particle exposed to a non-uniform and usually time-varying electric field. DC electric field can also apply the DEP force [16]. In this case, normally, insulators are used to apply a non-uniform electric field [17]. Depending on the electric properties of a particle and environment (buffer), particles are attracted to high-intensity electric field regions (positive DEP) or are repelled from them (negative DEP) [18]. Several methods, such as applying a negative DEP force [19], using the difference in cross-over frequencies (COF) [20], and using differences in sizes and polarization [21], have been investigated to modify the separation of healthy sperm and to increase fertility rates [22]. DEP has also been used for sex selection in recent studies; the effect of DEP on human sperm was examined and revealed that the DEP force was different on X and Y sperms due to the difference in the size of these sperms [23]. The difference in the COF of X and Y bovine sperms was also examined to improve sex selection [24]. One of the other applications of DEP is measuring the thrust force of moving cells when suspended in a media by equating the forward flagella thrust against negative DEP [25]. This method can also be used for measuring the force generated by the flagella of sperm. The forces imposed on the particle in a field generated by interdigitated electrodes numerically [26,27] and analytically using the Fourier series have been calculated [28]. Other DEP force measurement approaches include atomic force microscopy [29] and optical tweezers [30]. Measuring the accurate amount of the DEP force when imposed on sperm is essential to the design and optimization of the working conditions of actuators that use this phenomenon. The DEP force can be generally calculated by theoretical equations or numerical simulations. Scientists have always been concerned about the experimental measurement of the DEP force, as it is more accurate than numerical methods and can be used to validate them. One of the methods of the experimental DEP force measurement on a particle is to equate the DEP force with the gravity force inside a tilted channel [31] or with the drag force of the fluid [32]. In both methods, the friction force affects the measurements. Based on our search of the literature, it is of note that nearly all these studies have a neglected friction force. The results of this investigation aim to reveal that the friction force is not negligible in actuators that use a positive DEP force. Therefore, a method must be proposed to measure the DEP force by considering friction. So far, no such method has been described, and in this study the forces acting on the sperm in a positive DEP process were presented, taking into account the friction force.

## 2. Materials and Methods

### 2.1. Theory and Modeling

An electrically polarizable particle may form a dipole when placed in an electric field. If the electric field applied to the particle is uniform, the net force applied to the particle is zero. However, if the particle is located in a non-uniform electric field, the applied force to the particle can be obtained according to Equation (1) [33] as:

(1) $$F_{DEP}=(p.\nabla )E$$

In the above equation, *P* is the induced dipole moment of the particle due to the electric field, ∇ is the del vector differential operator used for vector transformation, and *E* is the electric field intensity.

If the particle is considered a sphere, after solving Laplace’s equation in terms of the boundary conditions of classical electrostatics and the vector transformation of $\left(p.\nabla \right)E$ in terms of *E*, the final result leads to the below equation [34].

(2) 〈FDEP〉=2πεmε0a3Re[KW]∇|E|2

In Equation (2) a, $\varepsilon_{m}$, $\varepsilon_{0}$, and $Re\left[K_{W}\right]$ represent the particle radius, the permittivity of the surrounding media, vacuum permittivity, and the real part of the Clausius–Mossotti factor, respectively. In case the particle is uniform, $K_{W}$ is given by

(3) $$K_{W}=\frac{\varepsilon_{p}^{*}-\varepsilon_{m}^{*}}{\varepsilon_{p}^{*}+2\varepsilon_{m}^{*}}$$

where $\varepsilon_{p}^{*}$ and $\varepsilon_{m}^{*}$ are the complex permittivity of the particle and media, respectively. $\varepsilon_{x}^{*}$ is determined by:

(4) $$\varepsilon_{x}^{*}=\varepsilon_{0}\varepsilon_{x}-j\left(\frac{\sigma_{x}}{\omega }\right)$$

Instead of “*x*” in the above equation, *p* (particle) or *m* (media) should be replaced to measure the complex permittivity ($\varepsilon_{x}^{*}$) and conductivity ($\sigma_{x}$) of the particle and media, respectively. $\varepsilon_{0}$ is the vacuum permittivity and equals $8.854\times 10^{-12}$ F/m, and ω=2πf (Rad/S) is the angular frequency. When the particle has a membrane layer, as most cells have, permittivity can be measured by the below equation:

(5) $$\varepsilon_{p}^{*}=\varepsilon_{mem}^{*}\frac{{\left(\frac{r+t}{r}\right)}^{3}+2\left(\frac{\varepsilon_{in}^{*}-\varepsilon_{mem}^{*}}{\varepsilon_{in}^{*}+2\varepsilon_{mem}^{*}}\right)}{{\left(\frac{r+t}{r}\right)}^{3}-\left(\frac{\varepsilon_{in}^{*}-\varepsilon_{mem}^{*}}{\varepsilon_{in}^{*}+2\varepsilon_{mem}^{*}}\right)}$$

where r and t are the cytoplasmic radius and cell membrane thickness, respectively. Additionally, $\varepsilon_{in}^{*}$ and $\varepsilon_{mem}^{*}$ are the complex permittivity of the cytoplasm and membrane.

As shown in Equation (3), the sign and value of ($K_{W}$) depend on the particle polarization and surrounding fluid, where a higher difference results in a higher force on the particle. The greater polarization of the particle compared to its surrounding media can lead to a positive Clausius–Mossotti factor and, consequently, a positive DEP force, which can direct the particle toward regions with the highest electric field gradient. The greater polarization of the fluid leads to a reverse phenomenon and negative DEP, in which the particle is repelled from regions with the highest electric field gradient.

In addition to the DEP force, gravity, drag, and buoyancy are other forces that a particle faces in a fluid. The cell-electrode adhesion force might affect the particle but was neglected in this research. The effect of other phenomena, such as heat-induced fluid flow, AC electroosmosis, and hydrodynamic lift forces, are explained later. The Stokes hydrodynamic drag force on a spherical particle can be calculated by Equation (6) [35].

(6) $$F_{drag}=6\pi \eta rv$$

where r is the hydrodynamic radius of the particle, v is the fluid flow velocity relative to the particle, and η is the viscosity of the medium.

**Figure 1:**
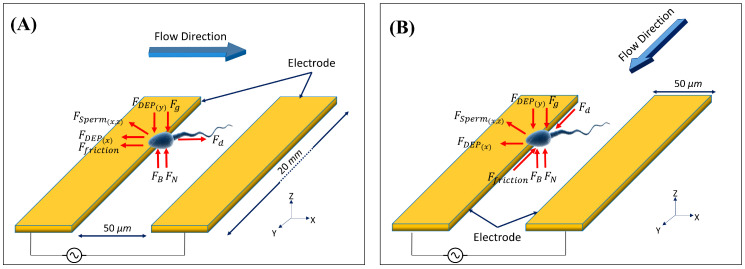
Imposed forces on sperm in fluid flow and positive DEP. (**A**) Fluid flow and electrodes in a perpendicular arrangement. (**B**) Fluid flow and electrodes in the same direction.

In separation actuators working with positive DEP, particles were attracted to the electrodes, separating them from other particles under a negative DEP effect. The drag force created due to the difference between the particle and fluid velocities could remove particles from the electrodes. The particles were carried away to the outlet if the fluid velocity surpassed a specific limit. The friction force also affected the particle removal process and prevented the particle from being washed up by the fluid flow. According to the above discussion, when a particle inside a fluid flow was subject to a non-uniform electric field, it faced different forces such as gravity, buoyancy, drag, surface friction, and DEP. Figure 1 illustrates the forces acting on sperm inside the fluid flow under the effect of positive DEP in two arrangements: perpendicular and parallel to the fluid flow.

The purpose of this study was to measure the DEP force experimentally. Equilibrium equations of the particle (sperm) were utilized. Writing the equilibrium equations in X and Z directions for the particle illustrated in Figure 1A gives Equations (7) and (8), respectively.

(7) $$X:{\left(F_{d}\right)}_{I}-F_{sperm}{}_{\left(x\right)}-F_{friction}-F_{DEP_{\left(x\right)}}=0$$

(8) $$Z:F_{B}+F_{N}+F_{DEP_{\left(z\right)}}+F_{Sperm_{\left(z\right)}}-F_{g}=0$$

In the above relations ${\left(F_{d}\right)}_{I}$, $F_{g}$, $F_{B}$, and $F_{sperm}$ are the drag force in the perpendicular arrangement of the channel and electrodes, gravity force, buoyancy force, and force induced by sperm, respectively. The drag force can be calculated by Equation (6) and the known values of fluid velocity and viscosity.

Since the fluid flow velocity is not the same in the cross-section of the channel and has a parabolic distribution, so, as a result, the drag force also varies at different heights of the channel [36]. To enhance the accuracy of the method, the drag force, which is proportional to the fluid velocity, was calculated by considering the location of the trapped particles.

The particles were trapped on the edge of the electrodes at the bottom of the channel. The particle’s radius was used as the desired location of the channel height to measure the fluid velocity (Figure S1 in the supplementary file).

The gravity force $F_{g}$ and buoyancy force $F_{B}$ were determined using the particle size and the density of the fluid and the particle. The force amount that a motile sperm generates, based on the literature, has been estimated to be in the order of 3 to 4 pN [37].

This study also used a simple simulation to calculate the drag force that a healthy sperm faces by COMSOL Multiphysics 5.2. Based on a previous study, a motile spermatozoa’s velocity is around 40 to 50 µm/s [38]. To numerically calculate the force generated by sperm, a particle similar to sperm dimensions and density can be placed inside a stationary fluid when its velocity reaches the velocity of the sperm, which is around 40 µm/s. According to our observation and others [39], the motility is reduced to nearly zero after 15 min of exposure to the DEP buffer; therefore, the sperm force is not sensible. Thus, we neglected this force in the tests.

The unknown parameters of the above equations are the vertical component of the DEP force $F_{DEP\left(z\right)}$, the horizontal component of the DEP force $F_{DEP\left(x\right)}$, the friction force $F_{friction}$, and the contact force $F_{N}$. Therefore, only using Equations (7) and (8) does not lead to a solution for these unknowns.

A usual method of solving such a problem in the literature is neglecting the friction force [31,32]. Thus, in the literature, one of the unknowns is reduced by neglecting the friction force, and consequently, the DEP force can be calculated based on this assumption. However, in this research, it was found that this assumption may not be accurate.

To address the above dearth, an alternative solution was implemented in our approach. By adding new equations to the above system, we resolved the problem that is shown in the experiment implemented in Figure 1. In this new approach, we repeated the experiment in Figure 1A by changing the flow direction from the *X*-axis to the *Y*-axis, as shown in Figure 1B. In the parallel arrangement of electrodes and the channel, particles were not affected by the DEP force in the flow direction (*Y*-axis) since the electric field gradient was zero in this direction and led to a zero value for the DEP force. Therefore, the friction force was the only force that prevented the particles from being moved away by the fluid flow. It should be noted that the setup, according to Figure 1B, is not suitable for separation as a lower force affects the particles. Therefore, in this study, the parallel arrangement was only used to measure the applied forces on a particle. The equilibrium equations in the Y direction for the particle shown in Figure 1B are as follows:

(9) $$Y:{\left(F_{d}\right)}_{II}-F_{friction}=0$$

In Equation (9), subscript “*II*” refers to the parallel arrangement of the channel and electrodes. The DEP force in parallel and perpendicular arrangements are identical since the applied voltage and frequency are equivalent.

The trapped location of the particles is also the same, as the electrodes are constant in both arrangements and only the fluid flow direction changes.

Thus, by replacing Equation (9) with Equation (7), the DEP force can be calculated by Equation (10). As a result, to calculate the DEP force, it is only necessary to apply equal voltages and frequencies to both experiments and then measure the minimum drag force that moves the particles. According to Equation (10), the difference between the drag forces in the parallel and perpendicular experiments (and also the sperm force, which is nearly zero in the DEP buffer) determines the horizontal component of the DEP force.

(10) $$F_{DEP_{\left(x\right)}}={\left(F_{d}\right)}_{I}-{\left(F_{d}\right)}_{II}-F_{sperm}{}_{\left(x\right)}$$

Although the sperm force amount was considered to be zero due to exposure in the DEP buffer, this was mentioned in the equations. Therefore, even with different DEP buffers that can preserve sperm motility, the above equations are applicable.

Hydrodynamic lift forces, heat-induced fluid flow, and AC electroosmosis are other phenomena whose effects should be discussed.

Since the cells attach to the channel’s bottom surface, hydrodynamic lift force (wall-induced lift force) also affects them [40]. Considering the parabolic fluid velocity distribution, the relative velocity amount is lower on the surface; therefore, the pressure is higher. As a result, the lift force pushes the cells to the channel centerline [41]. This force can be applied to the cells in the Z direction, affecting $F_{N}$ and, consequently, the friction. As the effect of friction has been considered in these experiments, hydrodynamic lift force was also considered.

The AC-Electroosmosis effect is dependent on the applied frequency. In high frequencies (>100 KHz), there is not enough time for the formation of an electric double layer [42]. In our case, the frequency was high enough (more than 800 KHz) to prevent an electric double-layer formation. Therefore, the effect of AC-Electroosmosis can be disregarded. Joule heating, which leads to a fluid flow motion, mostly depends on the conductivity of the media and the applied voltage. As a result, its effect is more significant in high-conductivity media (more than 100 mS/m) [43]. In this research, as the conductivity was not so high and there was also fluid flow inside the channel, this helped minimize the temperature rise, and the effect was neglected.

### 2.2. The Geometry of the Electrodes and the Microchannel

The electrodes used for generating the electric field and the microchannel were designed according to Figure S2 in the supplementary material. The electrode gap and also width of the electrodes were 50 μm. The thickness of the electrode was 120 nm.

The microchannel was made of polydimethylsiloxane (PDMS). The microchannel width was determined after several trials and tests (150 μm, 450 μm, and 1.1 mm). For the final test, the width was chosen to be 1.1 mm as this was large enough to prevent the microchannel from being clogged by the sperm. The height and length of the microchannel were 100 μm and 2 cm, respectively.

Since the height of the channel was 1000 times the thickness of the electrodes, the electrodes did not significantly affect the laminar flow. Figure S2 also shows the microchannel and the electrodes in parallel and perpendicular arrangements.

### 2.3. Simulation

In addition to the DEP force measurement, the finite element method implemented in COMSOL Multiphysics 5.2 was used to simulate the DEP force on WBC to verify the results, which will also be explained later. The simulation was performed in two dimensions since the geometry and boundary conditions did not change along the width of the microchannel. The electrical and mechanical properties of the simulated particle and fluid are presented in Table 1. Boundary conditions and dimensions are shown in Figure S3.

Particle size and concentration were low enough not to affect the electric field distribution. So, in the simulation, it was considered that the particles did not stick to each other to prevent any changes in the electric field distribution.

The domain was meshed by controlled tetrahedral elements. A denser mesh was used at the bottom of the microchannel because this had a higher electric field gradient than the upper region. However, the mesh along the microchannel was uniform.

### 2.4. Cell and Device Preparation

Two cell types, human sperm and WBC (Jurkat cells), were used to investigate and validate the results. Initially, individuals were informed about the aim of our study and its possible future prospects. The consent form was signed by each individual attending the Isfahan Fertility & Infertility Center and who donated their remaining semen sample for this study. For human sperm, fresh semen was obtained from eight donors (n=8). To calculate the DEP force, experiments on each donner’s sperm were repeated two times to reduce the errors in the measurements.

This study was approved by the ethical committee of the Royan Institute, with the code IR.ACECR.ROYAN.REC.1400.064. The WBCs were obtained from the cell bank of Tarbiat Modares University, Tehran, Iran.

As the semen environment is highly nutritious with high electrical conductivity, it cannot be directly used in the DEP process. Therefore, a buffer with lower conductivity was made and used. Additionally, as sperm are heterogeneous, each sperm has different properties. To solve this issue, the swim-up sperm separation method [44] was used to separate the highly mobile and healthy sperm in the hope of having a more homogenous sperm population.

After the preparation of sperm with the swim-up technique, the prepared sample was centrifuged, the supernatant was discarded, and the DEP medium was added to the pellet to minimize the number of electrolytes and residual albumin interfering with the DEP procedure. The final sperm concentration of each experiment was set to 1–2 million/mL by the DEP solution, and conductivity was set to 140 µS/cm. The sperm DEP buffer comprised 20 cc of deionized water, 1.7 gr of sucrose, and 0.06 gr of dextrose.

The DEP buffer of WBC was similar to the sperm DEP buffer, but the conductivity increased to 800 µS/cm by adding Phosphate buffered saline (PBS). WBCs were centrifuged two times. The final WBC concentration was set to 1–2 million/mL in the DEP solution.

Table 1 presents the properties of the sperm, WBC, and buffers used in the experiment and numerical simulation. The sperm were heterogeneous, i.e., their dimensions varied from sample to sample. Additionally, because of their ellipsoid shape, the surface area that faced the drag force was different in the perpendicular and parallel arrangements. Therefore, in the simulation, it was considered to be a sphere with a radius of 2.5 μm [45]. The microchannel and the glass surface were pre-treated with Polyvinyl Alcohol (PVA) (Sigma-Aldrich Corp., St. Louis, MO, USA) at a 1 mg/mL concentration for 30 min to prevent the sperm from sticking to them. Then, the microchannel was filled with a DEP buffer, and sperm was added through a syringe pump.

**Table 1:** Specifications of the WBC, sperm, and their buffers.

Properties	WBC Buffer	Sperm Buffer	WBC	Sperm	Reference
Working frequency (MHz)	-	-	0.8	1	-
Radius * (µm)	-	-	3.8	2.5	[45,46]
Membrane thickness (nm)	-	-	7	NK **	[47]
Permittivity	78	78	104	NK **	[46]
Membrane permittivity	-	-	12	NK **	[48]
Conductivity (µS/cm)	800	140	650	NK **	[46]
Membrane conductivity (nS/cm)	-	-	10	NK **	[46]
Density (kg/m^3^)	-	-	1019	1100	[49,50]
Viscosity (mPa.s)	1.2	1.2	-	-	[51]

* particle median radius, ** NK: Not known.

### 2.5. Fabricating of the Electrodes and the Microchannel

To make the electrodes, first, a thin layer of chromium, 20 nm thick, was deposited on the glass substrate, and then a 100 nm layer of gold was deposited on top of the chromium. Gold was chosen because of its electrochemical stability and biocompatibility [52]. The physical vapor deposition (PVD) method coated the gold and chromium layers. Then, a photoresist was coated on the gold layer by the spin coating method, and photolithography was used to create the desired pattern on the layer. Finally, the gold and chromium layers were etched, and the photoresist layer was removed.

To make the microchannel, after washing the silicon wafer substrate, the SU-8 photoresist was deposited by the spin coating method. Then, photolithography was utilized to create the desired microchannel pattern on the layer. Finally, the mold required for PDMS casting was made by developing the photoresist.

Two 2 mm diameter holes at the center of the inlet and outlet of the microchannel were punched to mount the Teflon tubes. Placing the manufactured microchannel on the electrodes was the last step in preparing the device for testing.

**Figure 2:**
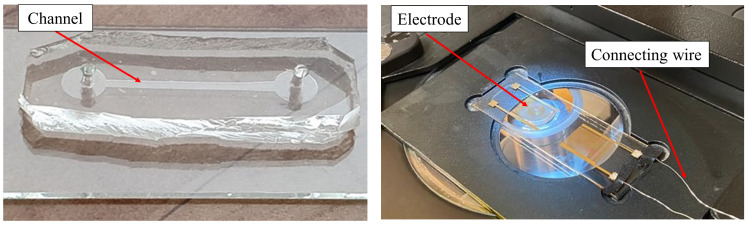
Manufactured electrodes and microchannel.

After fabricating the electrodes and microchannel, the geometry and dimensions of the created pattern were examined under a microscope. Figure 2 illustrates the electrodes and microchannel fabricated at the Sharif University of Technology.

### 2.6. Laboratory Setup

**Figure 3:**
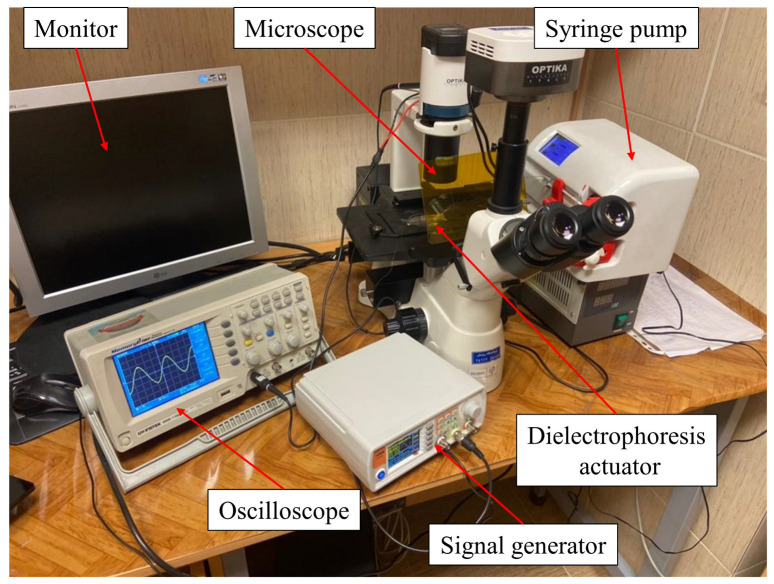
Experimental setup.

Figure 3 shows the laboratory setup used for the experiments. This setup included a syringe pump to provide a controlled fluid flow rate, a microscope for observation and to control the measuring process, a function generator to apply the signal to the electrodes, a computer for observing the tests, an oscilloscope for measuring the output signals, and silicon connection tubes to transfer the fluid to the microfluidic chip. To experiment, a DEP buffer, which also contained the cells, was collected in a 1 mL plastic syringe and then injected into the microchannel by a syringe pump through the Teflon tubes. The flow rate of the syringe pump could be adjusted with an accuracy of 0.01 mL/h. The fluid flow velocity was calculated by the known flow rate and dimensions of the microchannel. The signal generator created the desired voltage with an accuracy of 0.1 V. An inverted microscope was used to monitor the DEP process, and the voltage at which the cells attracted the electrodes was determined.

## 3. Results and Discussion

### 3.1. Simulation Results

In numerical simulations, it is necessary to perform a mesh independency analysis to ensure that the mesh is integrated. For this analysis, the voltage of a point in the middle of the electrodes at 20 μm height was examined. At this point, the voltage did not change with the number of elements for the meshes with more than 1000 elements. Therefore, the simulation results with 2056 elements, which were mesh independent, are described in the following.

To compare and validate the results of the proposed model, the DEP force on WBC with its electrical properties was measured beforehand and was calculated by the finite element simulation. For this purpose, the fluid flow in the microchannel was modeled by a steady flow analysis. Next, the distribution of the potential and electric field under a known frequency was simulated using frequency analysis. Finally, using these analyses, various forces acting on the particles, including drag, gravity, and DEP, were simulated at different periods, and the particle pathway with time was calculated. Figure S4 in the supplementary material demonstrates the simulated electric potential distribution and the particle pathway. As it is clear in Figure S4, the pass-way of the particles is highly dependent on their first vertical location. When the particles are located at a higher height in the channel, they move at a greater distance before being absorbed by the electrodes, and the capture time is increased accordingly.

The microchannel length required to have a fully developed flow was investigated in this simulation, and it was found that after an initial 50 μm, the fluid flow reached a fully developed state. Therefore, the force measurements and calculations in the simulation and experiments were performed after this length.

### 3.2. Experimental DEP Force Measurement

**Figure 4:**
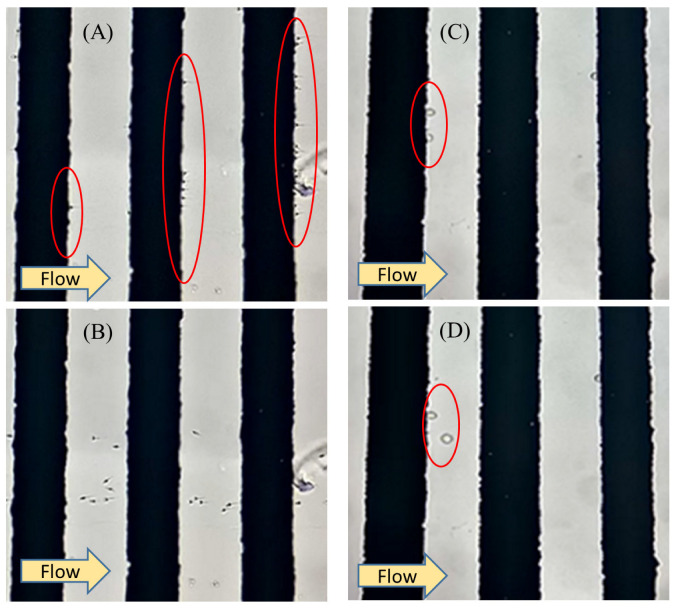
Trapping the sperm (1 MHz) and WBC (800 kHz) under the effect of positive DEP. (**A**) The applied voltage is high enough to prevent the trapped sperm from being washed by fluid flow. (**B**) The applied voltage is lower than the capture voltage, so the fluid flow removes the trapped sperm. (**C**) The applied voltage is high enough to prevent WBC from being washed by fluid flow. (**D**) The applied voltage is lower than the capture voltage, so the fluid flow removes the trapped WBC.

The voltage must be applied so that the cells are subjected to a positive DEP to attract them toward the electrode. In experimental tests, it was noted that 1 MHz was the proper frequency that led to positive DEP on the sperm. This frequency was determined by several experimental tests in which a favorable DEP force was applied to the sperm. 1 MHz was also reported in other works of research by applying a positive DEP on the sperm [53]. In these conditions, due to the positive DEP, sperm were attracted to the electrodes as the highest electric field gradient occurred on the edge of the electrodes. Figure 4A shows how the sperms were attracted and trapped by a positive DEP to the electrodes. The applied voltage and, as a result, the positive DEP force were both large enough that the fluid flow was unable to wash the sperm away from the electrode. In Figure 4B, by reducing the voltage, a positive DEP force on the sperm was reduced, and in this circumstance, the sperm was moved with fluid flow, which allowed it to exit the microchannel. Movies S1 and S2 in the Supplementary Material show how the fluid flow washed the sperms in perpendicular and parallel arrangements when the DEP force was set to zero.

The same test was performed on WBC; the results are presented in Figure 4C,D. Capture voltage is the minimum voltage so that the DEP force cannot prevent the cells from being washed away from the electrodes by the fluid flow.

**Figure 5:**
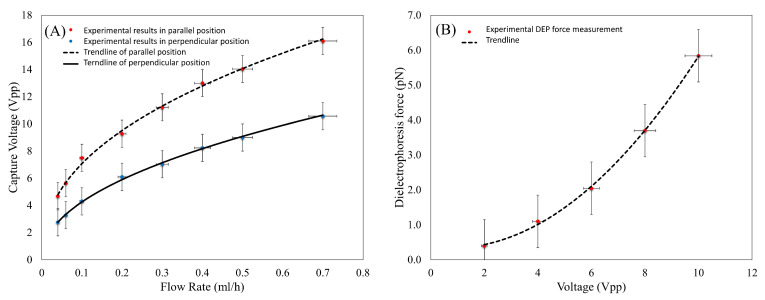
(**A**) Capture voltage of sperm at different flow rates. (**B**) Experimental measured DEP force imposed on sperm at different voltages.

Figure 5A demonstrates the effect of the fluid flow rate on the sperm capture voltage. The capture voltage increased by increasing the flow rate. A comparison between the trend lines for the parallel arrangement (in which the flow is on the *Y*-axis) with the perpendicular one (in which the flow is on the *X*-axis) revealed that the capture voltage was higher for the parallel arrangement. In other words, in the perpendicular arrangement, the DEP force was opposite to the drag force. Therefore, compared to the parallel arrangement in which the DEP force had no effect on the direction of the drag force, higher flow rates were needed to wash the cells in the perpendicular arrangement. This confirmed the discussion presented in the theory and modeling section.

According to Equation (9), the drag force exerted on the particle was equal to the friction force in the parallel arrangement of the microchannel and electrodes. So, the only force that prevented the cells from being washed was the friction force.

The friction force has been neglected in previous studies. However, as seen in Figure 5A, the friction force was considerable, and neglecting this force led to a significant error in the DEP force measurement.

The voltage effect of the DEP force acting on the sperm trapped at the bottom of the microchannel was examined using Equations (7)–(10), and the results are presented in Figure 5B. As shown, an increase in the applied voltage of the electrodes increased the force exerted on the sperm.

### 3.3. Validation

**Figure 6:**
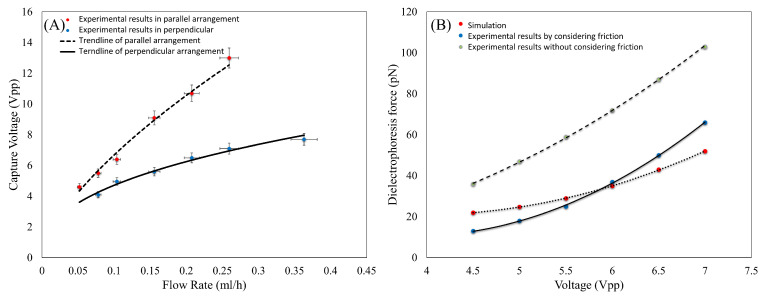
(**A**) Capture voltage of WBC in different flow rates. (**B**) Experimental and simulation results of DEP force imposed on WBC at different voltages.

To validate the new proposed model, the force measurement test on WBC, for which its electrical properties are known (Table 1), was repeated, and the DEP force on this cell was experimentally measured. The flow rate effect on the capture voltage of WBC is shown in Figure 6A under parallel and perpendicular arrangements. As can be seen, similar to the sperm, the capture voltage of WBC was also lower in the perpendicular arrangement than the captured voltage in the parallel arrangement.

Then, to validate the measurements, the electric field and fluid flow were simulated using the known electrical properties of the WBC [46]. These properties were used to calculate the force on the WBC in the simulation and finally compare it with the experimental data. Figure 6B compares the conventional method of the DEP force measurement by neglecting the friction force and the proposed model in this study, considering the friction force and simulation results. As can be seen, ignoring the friction force leads to a significant error in calculating the DEP force. This error becomes greater with an increase in the applied voltage. This clearly shows how much the presented model can improve the error related to the measurements. As it is clear, the simulation results reasonably matched the experimental data of the new method.

## 4. Conclusions

In this research, by implementing a new method for the DEP force measurement and using the experimental results, it was shown how neglecting the friction force leads to a significant error in measuring the DEP force on the cells. To eliminate this error, the governing equations of the DEP phenomenon were examined, and a new method independent of the cell type was proposed for measuring the DEP force by considering the friction effect. The proposed method is based on measuring the drag force and capture voltage under two experiments: when the fluid flow is parallel and perpendicular to the electrodes. In the experimental tests, the DEP force on human sperm and WBC was measured. The experimental results indicated that an increase in the fluid flow rate also increases the capture voltage of the cells. Since the electrical properties of WBC were known, the applied DEP force on WBC was numerically simulated and measured to validate the experimental results. The comparison between the simulation results, the conventional method, and the newly proposed method revealed that in the conventional method, the measured force could be up to twice the real force due to neglecting the friction effect. The source of this error was removed in the presented method. Therefore, the DEP force measurement accuracy was improved. Measuring the DEP force amount on human sperm by considering its ellipsoid shape could be suggested as a future topic.

## Data Availability

The data that support the findings of this study are available from the corresponding authors upon reasonable request.

## Supplementary Materials

The following supporting information can be downloaded at: https://www.mdpi.com/article/10.3390/bios13050540/s1, Supplementary equation: Equation (S1): Fluid flow distribution in the microchannel, and Supplementary figures: Figure S1: Schematic view of the microchannel, Figure S2: Electrodes and channel in (A) parallel and (B) perpendicular arrangements, Figure S3: Simulation boundary conditions, Figure S4: Simulation results (A) Electric Potential and (B) particle pathway in Vpp = 8 V and fluid velocity of 0.6 mm/s, and Supplementary movies: Movie S1: Trapping the sperms by DEP force in the flow rate of 0.1 mL/h and then letting them move away by setting the voltage to 0 (Perpendicular arrangement), Movie S2: Trapping the sperms by DEP force in the flow rate of 0.02 mL/h and then letting them move away by setting the voltage to 0 (Parallel arrangement).
