# Supplementary material for: Enhancing the Accuracy of Measuring DEP Force Applied on Cells by Considering the Friction Effect
Source: Biosensors (Basel). 2023 May 12;13(5):540. doi: 10.3390/bios13050540 (PMC10216049; doi:10.3390/bios13050540)
Supplement: Supplementary file 1 [file biosensors-13-00540-s001.zip › biosensors-2318268-supplementary.pdf]

Supplementary Materials

Enhancing the Accuracy of Measuring DEP Force Applied on Cells by Considering the Friction Effect

Alireza Khouzestani [1], Yousef Hojjat [1,*], Marziyeh Tavalaee [2], Hesam Sadeghian [1] and Mohammad Hossein Nasr-Esfahani [2,3,*]

[1] Department of Mechanical Engineering, Tarbiat Modares University, Tehran 14115-175, Iran; a.khouzestani@modares.ac.ir (A.K.); hesam.sadeghian@modares.ac.ir (H.S.)
[2] Department of Animal Biotechnology, Reproductive Biomedicine Research Center, Royan Institute for Biotechnology, ACECR, Isfahan 8165131378, Iran; m.tavalaee@royan-rc.ac.ir
[3] Isfahan Fertility and Infertility Center, Isfahan 8158858151, Iran
* Correspondence: yhojjat@modares.ac.ir (Y.H.); mh.nasr-esfahani@royaninstitute.org (M.H.N.-E.)

1. Fluid Flow Distribution in the Microchannel

Figure S1 presents a schematic view of a microchannel with a cell inside.

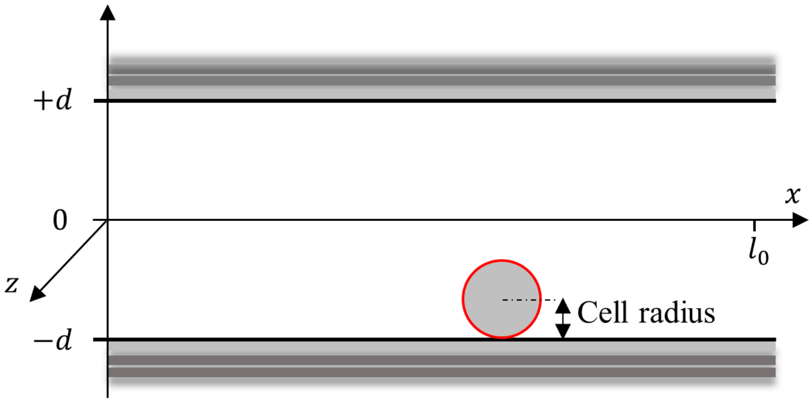

Figure S1. Schematic view of the microchannel.

By solving the Navier Stokes equations, Fluid flow velocity distribution in a rectangular microchannel is described in equation (S1) [1]:

$$U_x = \frac{1}{2\eta}\frac{p_0}{l_0}\left(d^2 - y^2\right) \tag{S1}$$

p_0 and η are inlet pressure and fluid viscosity. $2d$, w, and l_0 are the height, width, and length of the microchannel, respectively. Also, the microchannel width in the z direction is much larger than its height ($w \gg 2d$). Equation (1) indicates that a parabolic flow develops across the channel height.

On the wall of the microchannel, where $y = d$, fluid velocity will be zero. The maximum velocity is obtained in the middle of the channel, where $y = 0$.

For calculating the fluid velocity in the microchannel cross-section, the radius of the cells was considered as the height location, which was 3.6 μm for WBC and 2.5 μm for sperm. So, the drag force on the cells was measured by assessing the fluid velocity in the mentioned location.

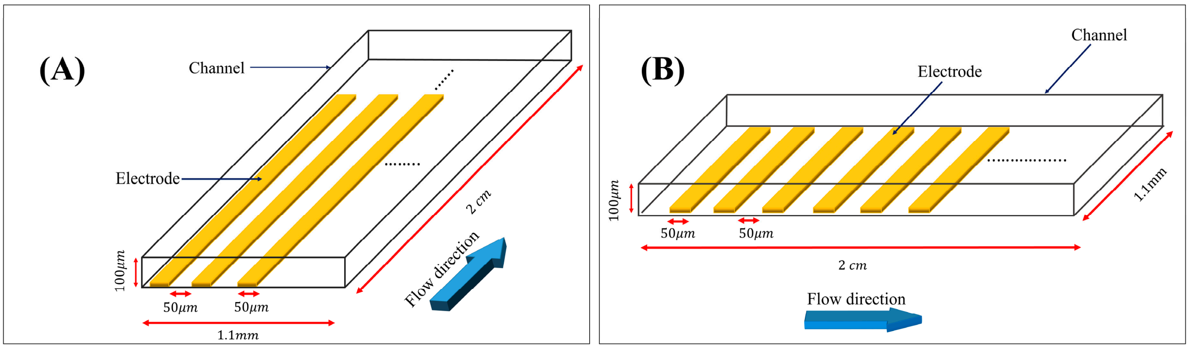

Figure S2. Electrodes and channel in (A) parallel and (B) perpendicular arrangements.

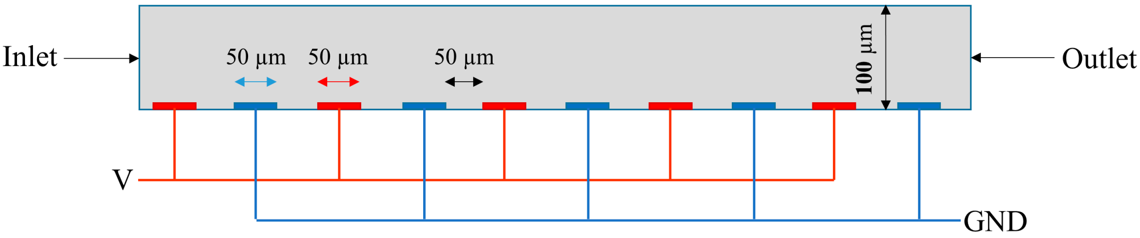

Figure S3. Simulation boundary conditions.

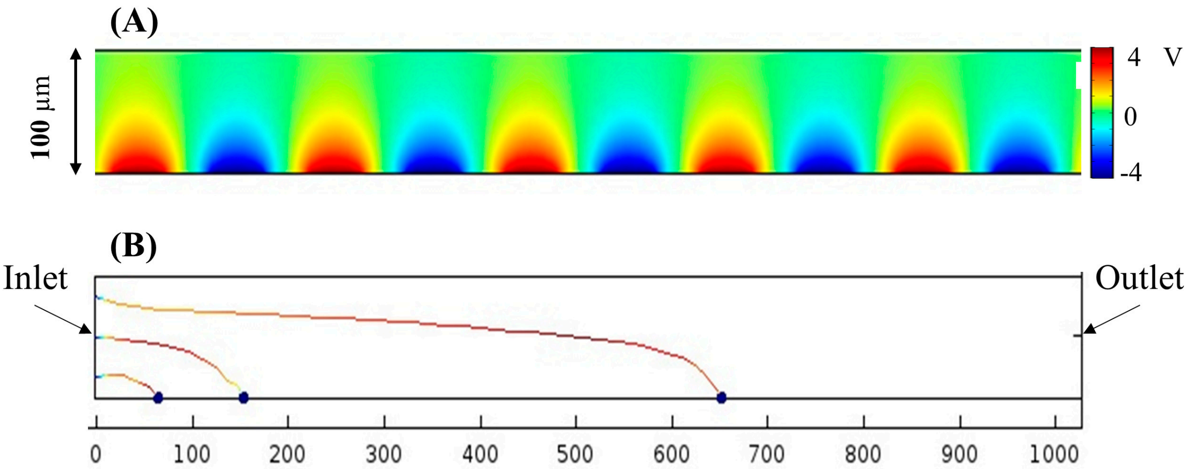

Figure S4. Simulation results (A) Electric Potential and (B) particle pathway in Vpp = 8 V and fluid velocity of 0.6 mm/s.

Supplementary Movies

Movie S1. Trapping the sperms by DEP force in the flow rate of 0.1 ml/h and then letting them move away by setting the voltage to 0 (Perpendicular arrangement).

Movie S2. Trapping the sperms by DEP force in the flow rate of 0.02 ml/h and then letting them move away by setting the voltage to 0 (Parallel arrangement).

Reference

1. 36. Bruus, H. Lecture Notes Theoretical Microfluidics. *Physics* **2008,** *18*, 363.
